# The Russian Union of Zemstvos: A Fine Organisation for the Care of the Wounded

**Published:** 1917-02-17

**Authors:** 


### THE RUSSIAN UNION OF ZEMSTVOS.

### A Fine Organisation for the Care of the Wounded.

Technically the word "Zemstvo" means a Rural County Council, and thus the Russian Union of Zemstvos is equivalent, as generally understood in England, to a Russian Union of Rural County Councils. This we learn from a preface to a report of the London Committee of the Union, but when we read further into the pages of this document it is evident that during the war this organisation, provided with State and charitable funds, possesses in a large measure the functions and powers, in Russia, nearly equal to those of our British Red Cross, Army Service Corps, and R.A.M.C. In registration, through local tribunals and in other ways the Government of this country have realised to what important use the organisation of our municipal bodies may be put; as far back as the time of the Russo-Japanese War our great Eastern Ally appreciated the value of established organisation on a large scale and made use of the municipal bodies in a national emergency. Then a certain number of Zemstvos joined together and offered the Government a part of the money raised by local taxation, together with their active help in the work of relief.

When war was imminent in the last days of July 1914 the same help was forthcoming, and on the initiative of the Zemstvo of Moscow a special committee was formed who evolved a scheme of a Union of Zemstvos for medical and sanitary purposes connected with the war. This work of mercy started with a substantial bank balance, as funds amounting to about £100,000, unexpended during the Japanese War, were given over to the Union's fund and, Imperial sanction being received, the work of the Union was taken under the flag of the Red Cross Society, while it preserved full independence.

### The Primitive Plan of the Union.

The primitive plan was for the Union to devote itself exclusively to the relief of the sick and wounded brought from the fronts into the interior of the country. The part thus intended to be played was subordinate to the sanitary organisation of the Ministry of War and of the Red Cross Society, and the Union was not allowed to work independently for the evacuation of the wounded or to extend its activity to the battle-line. According to these plans, and in consideration of the very limited funds, provision was made for about 25,000 to 30,000 beds and for a few hospital trains, but it soon became evident that many needs, some of them not directly connected with the relief of the wounded, had not been foreseen in peace-time, for the War Office and the Red Cross Society had to devote all their energy to the medical work at the Front. Thus was created the necessity of erecting numerous hospitals and of collecting and distributing centres for the sick and wounded coming from the Front, of providing adequate medical staffs and the equipment as well as trains for the transport of the wounded. All these pressing needs could be met only by a close alliance between the Government and the public corporations, of which the Union of Zemstvos, possessing the confidence of all classes and having at its disposal a well-trained staff, took the lead. Gradually the High Command laid on the Union the most varied and extensive tasks, including amongst many others the organising of a net of canteens, medical institutions, registration and labour offices, the relief of refugees, workshops, and refuges for children.

### How the Great Work Grew.

But the Union was destined to fulfil still more important functions, such as the supervision of workmen digging trenches at the Front and mending roads, the acceptance, registration, and dispatch of the wounded to hospitals in the interior of the country, providing measures of a sanitary and hygienic nature, such as the supply of good drinking water for the army and prevention against the importation of infection into the interior of Russia, and undertaking the purchase of clothes and other supplies for the troops; in connection with the last-named service the Union turned out nearly 36,000,000 pieces of clothing for the commissariat during the first sixteen months of the war.

We who are so near the Western area of the war, and know the automatic regularity of the services dealing with the provision of supplies and the transport of wounded, are perhaps unable to appreciate fully the enormous difficulties prevailing on more distant fronts. To extend our vision the account of the work of the Union in the Caucasus is particularly helpful. Sometimes in these lonely mountain regions it is impossible to find any place of refuge for the wounded and the staff, and as the climate is so severe the use of light tents is out of the question, while the prompt building of places of shelter is equally impossible because of the absence of wood. In the circumstances heavy tents and a large number of special oil-stoves, which can be utilised for camp kitchens as well as for heating, must be transported. The wounded and the forage have to be carried by pack-animals along the mountain tracks, as ordinary means of conveyance are quite unfit for the purpose, and a special kind of pack-stretcher, quite unknown before the war, has had to be invented. Thus from the snow-fields of the mountains down towards the roadway (where it is often so hot that travelling during certain hours is impossible) the wounded soldiers are carried on the backs of pack-animals. Over the roadway until the next summit is reached they are conveyed in motor-cars or in wagons, and then across the summit on sleighs.

The work of the Union of Zemstvos proves what tremendous results may be achieved by uniting all the really active forces of a country. Its functions have grown far beyond the limits originally intended by the Government, and it has successfully brought into existence many enterprises which are acknowledged to be indispensable to the victorious conclusion of the war.

